# Quantitative Analysis of Cepharanthine in Plasma Based on Semiautomatic Microextraction by Packed Sorbent Combined with Liquid Chromatography

**DOI:** 10.1155/2014/695231

**Published:** 2014-02-16

**Authors:** Camille Desgrouas, Marc Desbordes, Jérôme Dormoi, Evelyne Ollivier, Daniel Parzy, Nicolas Taudon

**Affiliations:** ^1^UMR-MD3, Institut de Recherche Biomédicale des Armées, Faculté de Pharmacie, Aix-Marseille Université, 13385 Marseille, France; ^2^UMR-MD3, Laboratoire de Pharmacognosie et Ethnopharmacologie, Faculté de Pharmacie, Aix-Marseille Université, 13385 Marseille, France; ^3^Unité de Parasitologie, Institut de Recherche Biomédicale des Armées, BP 73, 91223 Brétigny-sur-Orge, France; ^4^Unité de Toxicologie Analytique, Institut de Recherche Biomédicale des Armées, BP 73, 91223 Brétigny-sur-Orge, France

## Abstract

The spread of *Plasmodium falciparum* resistance toward most of the used drugs requires new antimalarial compounds. Taking advantage of the biodiversity, the ethnopharmacological approach opens the way for the discovery and the characterization of potent original molecules. Previous works led to the selection of a bisbenzylisoquinoline, cepharanthine, extracted from *Stephania rotunda*, which is mainly present in Cambodia. A sensitive and selective liquid chromatography method has been developed for the determination of cepharanthine in mouse plasma. The method involved a semiautomated microextraction by packed sorbent (MEPS) using 4 mg of solid phase silica-C8 sorbent. LC separation was performed on a Kinetex XB-C18 column (2.6 *µ*m) with a mobile phase of acetonitrile containing formic acid and 10 mM ammonium formate buffer pH 3.5. Data were acquired at 282 nm with a diode array detector. The drug/internal standard peak area ratios were linked via linear relationships to plasma concentrations (75–2,000 ng/mL). Precision was below 5% and accuracy was 99.0–102%. Extraction recovery of cepharanthine was 56–58%. The method was successfully used to determine the pharmacokinetic profile of cepharanthine in healthy and *Plasmodium berghei* infected mice. The infection did not impact pharmacokinetic parameters of cepharanthine.

## 1. Introduction

Affecting about 216 million and killing 660,000 people in 2010, malaria is still one of the most widespread parasitic tropical diseases. Its main impact is located in sub-Saharan Africa, where at least 90% of all malaria-related deaths occur [1]. The emergence of *Plasmodium falciparum* resistance to many antimalarial drugs is a severe problem all over the world. In front of the global spread of resistance, the WHO officially recommended in 2006 that artemisinin combination therapies (ACTs) must be adopted as first-line treatment of uncomplicated malaria caused by *P. falciparum* [2]. Unfortunately, recent reports from the South-East Asia revealed evidence of emerging resistance to artemisinin [3, 4]. If ACT efficacy should fail, no suitable alternatives exist as first-line treatments of *P. falciparum* malaria. If it had suddenly spread, this phenomenon would have catastrophic consequences, especially in Africa. Consequently, new antimalarial compounds, particularly those that are based on compounds structurally unrelated to existing antimalarial agents and that have new independent mechanisms of action, are needed in the battle against this major endemic disease. In front of this urgent need, the rich vegetable biodiversity, in particular in the tropical rainforests, is an important source of new chemical structures [5]. The majority of the world population relies mainly on natural products for their primary healthcare. Studying relationships between people and plants, ethnobotanists have a responsibility both to scientific community and to indigenous culture. An important number of modern drugs have been isolated from natural resources. Besides, it is particularly interesting to note that the emergence of resistance to antimalarial drugs isolated from plant might be slower to appear. For example, one year only after its marketing, resistance cases were reported for atovaquone. It took 15 years to observe a loss of efficacy with aminoquinolines. Quinine, isolated from *Cinchona,* is still useful despite more than 350 years of continuous use. Artemisinin's spot of resistance limited to the South-East of Asia has been described since the last few years, whereas *Artemisia annua*, from which artemisinin is extracted, was used for more than 2,000 years [6]. Thus, as source of new drugs and insecticides, interest in medical plants has increased in recent years. New chemical structures, among which some may be potentially helpful against infectious diseases like malaria, have been isolated but many still remain to be discovered. It is in this context that an inquiry was performed on Cambodian plants used in traditional medicines. An antiplasmodial activity was researched on extracts from 28 indigenous wild plant species. Twenty-six extracts from 8 plants showed an antiplasmodial activity. Among them, extracts from *Stephania rotunda *Lour. (Menispermaceae) possessed an interesting activity [7]. *Stephania rotunda* is a plant growing on calcareous cliff of Cambodian mountain areas [8]. Cheng and Huon mentioned *S. rotunda* to be active on malaria and fevers [9]. Concentrations at which extracts were able to inhibit 50% of parasitic growth (IC_50_) on the *P. falciparum *chloroquine-resistant strain W2 were, respectively, 1.0 and 2.8 *μ*g/mL for dichloromethane and water extracts of *S. rotunda* tuber [7]. Fractionation of dichloromethane extracts allowed the isolation of a bisbenzylisoquinoline named cepharanthine. Exerting an interesting activity on plasmodial culture (IC_50_ above 0.60 *μ*M on W2 strain) [10], efficacy of cepharanthine was tested in Balb/c mice infected by *P. berghei*. At the dose of 10 mg/kg administered by intraperitoneal and oral routes, cepharanthine induced a decrease of the parasitemia by 47% and 50%, respectively [10]. The present work involves the development of a semiautomated microextraction by packed sorbent (MEPS) for the extraction of cepharanthine in plasma sample. Previous works have reported analytical methods for the extraction and quantification of cepharanthine in plasma sample from beagle dog and human [11, 12]. However, no analytical methods have been reported for the quantification of cepharanthine in mouse, which is a classic malaria model. Moreover, in these two papers the method of sample clean-up was a protein precipitation (PPT) using solvents. PPT is a rapid, nonspecific method that can be utilized for sample cleanup in a high-throughput, automated manner. Nevertheless, the major limit of PPT is that many matrix components such as lipids, formulation agents, and substances remain in the supernatant after centrifugation. Thus, problems may occur including baseline noise, ghost peak, or carryover for example. In light of these considerations, the development of a solid-phase extraction was emphasized. Moreover, in the context of the study presented here, low volumes of plasma samples from mouse were collected. Thus, the purification and concentration of analytes using MEPS to clean up samples seemed relevant [13, 14]. In addition, this step of pretreatment was semiautomated. The sample cleanup and analyte concentration by the MEPS process allowed the quantification of cepharanthine in low volume of sample by a LC-DAD method. This method was validated according to validation procedures, parameters, and acceptance criteria [15–17]. It was used to analyze samples from a preclinical study performed for the first time in the mouse. Pharmacokinetic results in healthy and infected animals are presented.

## 2. Experimental

### 2.1. Chemicals and Reagents

Cepharanthine was isolated and purified from *Stephania rotunda *Lour. tuber by the Laboratory of Pharmacognosy and Ethnopharmacology (UMR-MD3, Aix-Marseille University) with identification and purity evaluated by elemental analysis greater than 99.6%. Berberine chloride form was obtained from Sigma (St. Louis, MO, USA). All solvents and chemicals were of analytical grade. Acetonitrile, methanol, ammonium formate, and formic acid were purchased from Fischer Scientific SAS (Illkirch, France). The formate buffer (630 mg/L ammonium formate) was prepared in purified water and adjusted to pH 3.5 with formic acid. In-house deionized water was further purified with a PURELAB Ultra system (ELGA LabWater, Antony, France). Polyoxyethylated 12-hydroxystearic acid (Solutol HS 15) was obtained from BASF (Ludwigshafen, Germany). For method validation, human plasma was obtained from pooled plasma samples collected from healthy volunteers not undergoing drug therapy (Etablissement Français du Sang, Marseille, France). Plasma samples from BALB/c mice (Charles River Laboratories, L'Arbresle Cedex, France) were collected with sodium heparin. The blood was centrifuged at 3,000 ×g for 10 min to obtain plasma. The drug-free plasma was aliquoted, stored at −80°C, and then used during the study in the preparation of quality control (QC) samples.

### 2.2. Instrumentation and Chromatographic Conditions

Optimization of various experimental parameters including the nature of the stationary phase, composition of the eluent, nature of the organic modifier, and temperature of the column was carried out (data not shown). The chromatographic analysis was performed using a Waters Acquity UPLC instrument (Milford, MA, USA). Data acquisition and processing were performed using Water's Empower 2 software (Milford, MA, USA). Separation was carried out on a Kinetex XB-C18 column (100 mm × 4.6 mm, 2.6 *μ*m, Phenomenex, Torrance, CA, USA) maintained at 40°C. The mobile phase consisting of 10 mM formate buffer pH 3.5—acetonitrile with 0.5% formic acid (74 : 26, v/v) was delivered isocratically at a flow rate of 0.6 mL/min. The detector wavelength was fixed at 282 nm. As it was previously published, chelidonine was used as internal standard (IS) [18]. Unfortunately, extraction difficulties occurred. Thus, berberine, another natural alkaloid with closely related chemical structure, was used. The chromatographic run time was 9 minutes.

### 2.3. Preparation of Standards and Quality Control (QC) Samples

Cepharanthine was dissolved in methanol to prepare a primary stock solution at a concentration of 1 mg/mL. The IS primary stock solution was prepared in purified water at a concentration of 1 mg/mL. Working solution was prepared by diluting extemporaneously the primary stock solution 1,000-fold in purified water. Calibration standards over the concentration range 75–2,000 ng/mL were prepared freshly by successive dilution of the primary stock solution in blank human plasma. One calibration curve includes a blank matrix and 7 calibration points. QC samples at three different levels were independently prepared at concentrations of 125 ng/mL, 300 ng/mL, and 1,500 ng/mL in blank mouse plasma. A blank mouse matrix was added too. After preparation, calibration standards and QC samples were vortexed for 10 seconds and incubated at 4°C in the dark for 30 min to allow a steady state with plasma components. The QC samples were prepared from a stock solution that was different from the one used to generate standard curves samples.

### 2.4. Sample Preparation Procedure

A semiautomated MEPS procedure was optimized [13, 14]. Optimization of various experimental parameters including the nature of the sorbent was carried out. Assays were performed with divinylbenzene copolymers (DVB and SDVB) and silica-based (C18 and C8) sorbent. Tests were performed with gas-tight syringe of 100 *μ*L and 250 *μ*L. Upstream extraction and effect of (i) protein precipitation with various volumes of water containing assorted trifluoroacetic or orthophosphoric acids and (ii) dilution with assorted volume of water or saturated sodium tetraborate solution have been tested. The BIN (Barrel Insert and Needle Assembly) washing step was optimized testing various combinations of solvents (water, methanol, acetonitrile, and isopropanol). The elution step has been optimized by evaluation of the efficacy of various volumes and combinations of acetonitrile with mobile phase. The conditions laid down for this study were the best compromise of signal-noise ratios at the retention time of the analytes. MEPS was carried out of a BIN containing 4 mg of solid phase silica-C8 sorbent (XCH 22 GA), inserted into a 100 *μ*L gas-tight syringe controlled by the automated analytical syringe eVol (SGE Analytical Science, Melbourne, Australia). Sample clean-up parameters were as follows. The sorbent was pre-washed with 3 × 50 *μ*L of methanol followed by 3 × 50 *μ*L of purified water before use. A volume of 75 *μ*L of plasma calibration standards and samples was treated with 25 *μ*L of the IS working solution (1 *μ*g/mL). The volume of 100 *μ*L of the mixture was drawn up and down through the syringe 10 times without discarding it. The sorbent was washed once with 30 *μ*L of a mixture of water and methanol (70 : 30, v/v); then the analytes were eluted with 30 *μ*L of 10 mM formate buffer pH 3.5—acetonitrile with 0.5% formic acid (74 : 26, v/v) and injected in the UPLC system. After each extraction, cleaning of the sorbent was done with 3 × 100 *μ*L of acetonitrile containing 0.5% of formic acid. At every step, the lower flow rate of the semiautomated syringe (3.33 *μ*L*·*s^−1^) was applied.

### 2.5. Data Analysis

Analyte-to-internal standard peak area ratios were used as the assay parameter. To define the relationship between peak area ratios and nominal cepharanthine concentrations, unweighted or weighted linear regression model (*Y* = *aX* + *b*), in which *Y* is the peak area ratio and *X* is the nominal concentration of the analyte, was tested. The regression curve was not forced through zero. The resulting equation parameters were used to calculate “back-calculated” concentrations for the calibrators. The good agreement between added and back-calculated concentrations was statistically evaluated. The normal distribution of the residuals (the difference between nominal and backcalculated concentrations) was verified. Moreover, the mean residual values were computed and compared to zero (Student's *t*-test); the 95% confidence interval was also determined.

### 2.6. Accuracy, Precision, Extraction Recovery, and Lower Limit of Quantitation (LLOQ)

Precision and accuracy were assessed by performing replicate analyses of QC samples at the abovementioned three concentrations against a calibration curve. The procedure was repeated on different days (*n* = 7) to determine interday accuracy and precision validation data. The percent relative standard deviation (RSD) served as the measure of precision. The accuracy was evaluated as [mean found concentration/nominal concentration] × 100. The extraction recoveries of cepharanthine over the QC ranges were determined by comparing peak areas of cepharanthine obtained from each QC sample prepared in plasma extracted as described above and those obtained from direct injection of a solution containing cepharanthine at the same concentrations dissolved in a mixture corresponding to the mobile phase described for the chromatographic conditions. The extraction recovery was also determined for the internal standard. The LLOQ was defined as the lowest concentration on the calibration curves for which acceptable accuracy (within 80–120%) and precision (≤20%) were obtained.

### 2.7. Stability

The stability of cepharanthine in mouse plasma was evaluated in reconstituted extracts (at 4°C in the autosampler for 120 h) and in the matrix (at 4°C for 120 h, and at −80°C for 12 months). Stability data are based on replicate determinations of QC samples and on replicate determinations of real sample from pharmacokinetic.

### 2.8. Application of the Method to a Pharmacokinetic Study in Healthy and *Plasmodium berghei *ANKA Infected Mice

Experiment was performed in 52 BALB/c female mice weighting 21.6 ± 1.3 g and purchased from Charles River (L' Arbresle Cedex, France). All animals were pathogen-free and were housed under standard conditions, with unlimited access to food and water. Animal welfare and experimental procedures were in accordance with the “Principles of Laboratory Animal Care” (NIH publication No.85-23, revised 1985) and with the “Guide for Care and Use of Laboratory Animals” and were approved by the local Animal Ethics Committee (Tropical Medicine Institute, Marseille, France). Prior to administrations of cepharanthine, 28 mice were infected by the rodent malaria parasite, *P. berghei *ANKA. Briefly, 24 mice were infected by intraperitoneal inoculation with 10^6^ of infected red blood cells from 4 donor mice, diluted in normal saline. The administration of cepharanthine was performed 3 days later, after individual confirmation of positive parasitemia by blood smear. Healthy and infected (*n* = 24 by group) mice received intraperitoneal administration of cepharanthine at the dose of 21 mg/kg. The solution for administration of cepharanthine was prepared freshly and consisted of 4.2 mg*·*mL^−1^ of cepharanthine in a mixture composed of methanol, water for injection, and Solutol (30 : 49 : 21, v/v/w). The dose was individually adjusted and based on 100 *μ*L for 20 g body weight. Blood samples (one sample per mice) were drawn in heparinized polypropylene tubes at the following timepoints (3 animals per time-point): before administration, 5, 15, and 30 min and 1, 2, 4, and 6 h after drug administration. Blood samples were collected by retroorbital bleeding in anesthetized animals with isoflurane (Mundipharma, Issy-les Moulineaux, France) and then centrifuged at 3,000 g for 10 min. Plasma samples were transferred into polypropylene tubes and stored at −80°C until assay. A blood smear was done for each sample coming from an infected mouse. At the end of the bleeding, mice were sacrificed by cervical dislocation. Noncompartmental analysis was performed using WinNonlin software version 6.3 (Pharsight Co., Mountain View, CA, USA). The area under the plasma concentration-time curve (AUC_0–6 h_) was calculated using the linear log trapezoidal method. The peak plasma concentration (*C*_max⁡_) and the time to reach the peak plasma concentration (*T*_max⁡_) were observed from the values of experimental data. The elimination rate constant (*K*_el_) was estimated by regression analysis from the slope of the line of best fit, and the elimination half-life (*t*_1/2_) of the drug was obtained by 0.693/*K*_el_. Total clearance (CL) and apparent volume of distribution (*V*_*d*_) were uncorrected for bioavailability (*F*).

## 3. Results and Discussion

### 3.1. Selectivity

**Figure 1:**
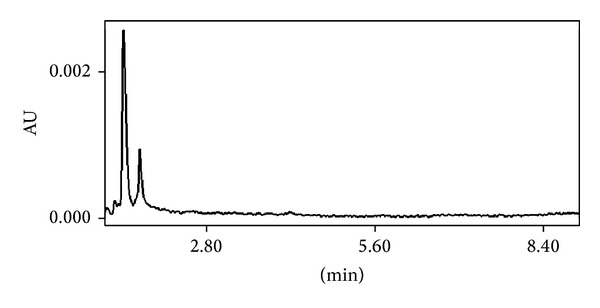
Representative chromatogram of blank plasma mouse after the MEPS procedure.

**Figure 2:**
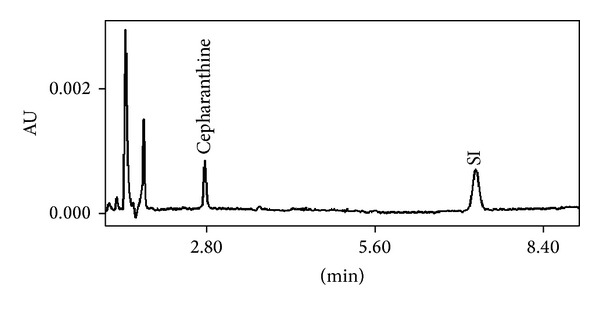
Representative chromatogram of a real sample from the pharmacokinetic study in mouse 4 hours after intraperitoneal administration of cepharanthine.

The analysis of blank matrices from different sources showed the absence of interfering endogenous components at the retention times of the cepharanthine and internal standard.  Figure 1 shows typical chromatograms from of blank plasma mouse. Representative chromatogram from real sample is shown in Figure 2. The specificity of this method was demonstrated by representative chromatograms of blank matrices, which indicated that each analyte was well resolved from the matrix endogenous peaks. No interference was found with any tested antimalarial drugs (i.e., atovaquone, chloroquine and its major metabolite desethylchloroquine, desethylamodiaquine, doxycycline, quinine, mefloquine, and primaquine).

### 3.2. Relationship between Response and Nominal Concentrations

Interassay repeatability was determined for calibration curves prepared on different days (*n* = 7). Observed retention times (*n* = 30) were 2.69 min for cepharanthine and 6.97 min for the internal standard (RSDs, 0.04–0.13%). For each point on the calibration curves, the concentrations were back calculated from the corresponding linear equation parameters. For concentrations of calibration standards, the RSD around the mean value does not exceed 10.5%. The goodness of fit between back-calculated concentrations and nominal concentrations was statistically evaluated (i) by comparing the regression line of back-calculated versus nominal concentrations to the reference line (slope = 1 and intercept = 0); no significant different was observed (slope = 1.02, 95% confidence interval (IC_95%_) [0.99, 1.04]; intercept = −8.52, IC_95%_ [−25.7, 8.62]); (ii) by studying the frequency distribution histogram of the residuals, which were normally distributed and centered around zero, the number of positive and negative values being approximately equal (Skewness = 0.43, IC_95%_ [−0.29, 1.14]; Kurtosis = 0.07, IC_95%_ [−1.33, 1.48]); (iii) by comparing the bias to zero (−1.735, IC_95%_ [−14.9, 11.4]); a *t*-test showed that this parameter was not statistically different from zero.

### 3.3. Accuracy, Precision, Extraction Recovery, and Lower Limit of Quantitation (LLOQ)

Precision was below 5% and accuracy ranged from 99.0 to 102.0%. Individual results are presented in Table 1. These data are well within the generally required validation criteria limits. The mean absolute extraction recoveries determined with seven replicates for each QC level were 57.8% (RSD, 11.0%) and 77.2% (RSD, 11.7%) for cepharanthine and internal standard, respectively. It was not statistically different over the range of concentrations studied. The lower limit of quantitation was 75 ng/mL. At this level, the precision of determination, expressed as RSD, was less than 11%, with adequate accuracy assay.

### 3.4. Stability

After a period of 120 h stored at 4°C, no degradation of cepharanthine was observed for either plasma samples or reconstituted extracts. There was no statistical difference between freshly prepared samples (*P* < 0.05) and back-calculated concentrations with values equal to 97.0 ± 5.0% and 99.5 ± 9.4%, respectively. In samples from pharmacokinetic study frozen at −80°C, cepharanthine was stable for at least 12 months. A paired comparison with concentrations previously observed did not show significant difference (*P* < 0.05).

### 3.5. Pharmacokinetic Study in Healthy and *Plasmodium berghei* ANKA Infected Mice

**Figure 3:**
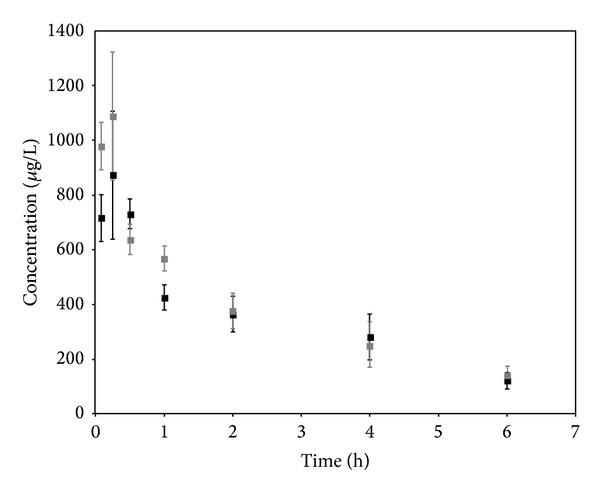
Mean plasma concentration-time curves of cepharanthine in healthy (bold square) and *Plasmodium berghei* ANKA infected mice (gray square) after intraperitoneal administration of cepharanthine at the dose of 21 mg/kg.

Semilogarithmic plots of the mean (±S.D.) cepharanthine plasma concentration-time profiles are illustrated in Figure 3. All the mice in the infected group presented a positive parasitemia at the time of drug administration (average = 10.4%; IC_95%_ [9.3, 11.5]). Maximum concentration was obtained 15 min after drug administration and was not statistically different between the two groups (*P* = 0.20). Pharmacokinetic parameters are summarized in Table 2. In the experimental conditions described above, pharmacokinetic parameters of cepharanthine were not influenced by the infection.

## 4. Conclusion

In the present paper, a semiautomated microextraction using packed sorbent method was developed to achieve cleanup of cepharanthine in low volume of plasma sample. Quantification of cepharanthine was performed with a LC-DAD method which was validated according to usual guidelines. This method offers an interesting alternative to previous study reporting applying LC MS/MS after a sample preparation based on a deproteinization by solvent protocols [11, 12]. Requiring low volume of sample, this method was successfully applied to the determination of cepharanthine pharmacokinetic parameters in healthy and malaria infected mice. Contrary to other antimalarial drugs [19, 20], it has been shown that pharmacokinetic parameters of cepharanthine did not seem to be impacted by the infection. Further studies targeting cepharanthine's metabolism pathways and the identification of metabolites could provide additional information.

## Figures and Tables

**Table 1:** Extraction recovery, precision, and accuracy of the method (*n* = 7).

Theoretical nominal concentration (ng·mL^−1^)	Extraction yield (%)	Accuracy (%)	Precision (%)
125	58.3	99.1	4.1
300	56.4	102	3.8
1500	58.8	99.0	4.2

**Table 2:** Pharmacokinetics parameters in healthy and *Plasmodium berghei* ANKA infected female BALB/c mice after a single intraperitoneal administration of cepharanthine at the dose of 21 mg/kg bodyweight (mean).

Parameters	Healthy mice	Infected mice
*T*_max⁡_ (h)	0.25	0.25
*C*_max⁡_ (*µ*g·L^−1^)	874	1.088
*t*_1/2elim_ (h)	2.52	2.59
*V*_*d*_ (L·kg^−1^)/F	31.8	30.1
Cl (L·h^−1^·kg^−1^)/F	8.77	8.04
AUC_(0–6 h)_ (*µ*g·h·L^−1^)	2069	2205
